# Supplementary material for: Economic burden of maternal morbidity – A systematic review of cost-of-illness studies
Source: PLoS One. 2020 Jan 16;15(1):e0227377. doi: 10.1371/journal.pone.0227377 (PMC6964978; doi:10.1371/journal.pone.0227377)
Supplement: S1 Table — (PDF) [file pone.0227377.s002.pdf]

S1 Table - Search Strategy

November 2017 Search strategy
#1 maternal:ti,ab OR maternity:ti,ab OR mother:ti,ab OR postpartum:ti,ab OR 'postpartum':ti,ab OR postnatal:ti,ab OR 'post-natal':ti,ab OR antenatal:ti,ab OR 'antenatal':ti,ab OR puerperal:ti,ab OR pregnan*:ti,ab OR childbirth:ti,ab OR nullipar*:ti,ab OR multipar*:ti,ab
#2 'maternal care'/exp
#3 'maternal health service'/exp
#4 MATERNAL HEALTH (OR/1-3)
#5 'cost of illness':ti,ab
#6 'cost analysis':ti,ab
#7 'health care cost':ti,ab
#8 'healthcare cost':ti,ab
#9 'economic burden':ti,ab
#10 'burden of illness':ti,ab
#11 'cost of illness'/exp
#12 COST OF ILLNESS (OR/26-32)
#13 #4 AND #12
[No time limits applied]
November 2019 Search strategy
#1 (cost* OR economic):ti
#2 (pregnan* OR matern* OR antenatal OR postpartum OR postnatal OR post-natal OR perinatal):ti
#3 #1 AND #2
[No time limits applied]
