# Supplementary material for: Economic burden of maternal morbidity – A systematic review of cost-of-illness studies
Source: PLoS One. 2020 Jan 16;15(1):e0227377. doi: 10.1371/journal.pone.0227377 (PMC6964978; doi:10.1371/journal.pone.0227377)
Supplement: S2 Table — (PDF) [file pone.0227377.s003.pdf]

S2 Table – Data extraction table for all included studies

Author	Year	Morbidity	Setting	Study group	Ns	Comparison group	Nc	Period of data collection	Accrual period for costs	Included costs	Base year for costs	Perspective	Discounting	Study design	Results (absolute cost increase, percentage increase)
Chen et al.[1]	2009	Diabetes	US	Pregnant women with gestational diabetes	N/A	Pregnant women without gestational diabetes	N/A	2003-2005	During pregnancy and 12 months postpartum	Mothers and children	2007	Payer	No	Modelling	GDM associated with incremental cost of €3,509 (%NR) per birth.
Kolu et al.[2]	2011	Diabetes	Finland	Pregnant women without gestational diabetes (GDM) or risk factors	35589	Pregnant women without GDM but with risk factors, GDM but no risk factors, and both GDM and risk factors	20547	2006	During pregnancy only	Mothers only	2006	Payer	No	Cross-sectional	All groups associated with higher mean antenatal care costs (-GDM/+risk factors: €174, 10%; +GDM/-Risk Factors: €406, 24%; +GDM/+Risk Factors: €682, 40%)
Cavassini et al.[3]	2012	Diabetes	Brazil	Inpatient and outpatient pregnant women (of any age) with diabetes or mild hyperglycemia	50	Pregnant teenagers without diabetes or hyperglycaemia	18	2007-2008	During pregnancy and birth	Mothers and neonatal care	2009	Payer	No	Cross-sectional	Total additional cost attributable to diabetes was €3,920 (161%) for inpatients and €185 (8%) for outpatients
Kolu et al.[4]	2012	Diabetes	Finland	Pregnant women at high risk of GDM and a diagnosis of GDM	251	Pregnant women at high risk of GDM without a diagnosis of GDM	597	2007-2009	From 12 weeks gestation to discharge from hospital after birth	Mothers and neonatal care	2008*	Societal	No	Cross-sectional	GDM associated with significantly higher total costs of care (€1,468, 25%)
Anderberg et al.[5]	2012	Diabetes	Sweden	Pregnant women with gestational diabetes	488	Pregnant women without gestational diabetes	950	1998-2009	10-14 years after the birth	Mothers only	2009	Payer	No	Longitudinal	Average increase annual total cost for those with GDM at end of 14-year follow-up was €401 (39%, not statistically significant)
Gillespie et al.[6]	2013	Diabetes	Ireland	Pregnant women with gestational diabetes	354	Pregnant women without gestational diabetes	4018	2006-2009	During pregnancy and birth	Mothers and neonatal care	2009*	Payer	No	Cross-sectional	GDM associated with significantly higher total unadjusted costs of care (€2,313, 51%)
Son et al.[7]	2014	Diabetes	South Korea	Pregnant women without diabetes	1171575	Pregnant women with gestational diabetes or pre-existing diabetes	110923	2010-2012	During pregnancy and birth	Mothers only	2011*	Payer	No	Cross-sectional	Both GDM (€263, 11%) and pre-existing DM (€672, 27%) were associated with significantly higher costs
Dall et al.[8]	2014	Diabetes	US	Mothers with gestational diabetes	N/A	Mothers without gestational diabetes	N/A	2012	Annual costs during year of birth	Mothers only	2012	Societal	No	Modelling	Excess annual costs associated with gestational diabetes was €4,893 (%NR) per woman

Author	Year	Morbidity	Setting	Study group	Ns	Comparison group	Nc	Period of data collection	Accrual period for costs	Included costs	Base year for costs	Perspective	Discounting	Study design	Results (absolute cost increase, percentage increase)
Danyliv et al.[9]	2015	Diabetes	Ireland	Pregnant women with gestational diabetes	270	Pregnant women without gestational diabetes	388	2007-2010	During pregnancy and annual postpartum costs	Mothers and neonatal care	2012	Payer	No	Cross-sectional	GDM associated with increased cost of birth (€865, 15%) and annual care costs postpartum (€720, 133%). Equivalent to incremental cost of €1,584 (25%) from birth to 12 months postpartum
Jovanovic et al.[10]	2015	Diabetes	US	Insured pregnant women with T1DM, T2DM, GDM, progressing GDM	58320	Insured pregnant women without diabetes	586875	2005-2011	Pregnancy and 3 months postpartum	Mothers and children	2008*	Payer	No	Cross-sectional	Significant increase in cost for all types of diabetes (T1DM: €12,561, 92%; T2DM: €7,993, 58%; GDM: €3,263, 24%,and Progressing GDM: €8,294, 61%).
Whiteman et al.[11]	2015	Diabetes & Obesity	US	Pregnant women who were overweight/obese, with and without gestational diabetes	51997	Normal weight pregnant women without gestational diabetes	524846	2004-2009	Birth and 12 months postpartum	Mothers and children	2010	Payer	No	Cross-sectional	Being overweight/obese alone (€774, 11%) and being overweight/obese with GDM (€1,426, 20%) were associated with higher average costs of maternal and infant care
Lenoir-Wijnkoop et al.[12]	2015	Diabetes & Obesity	US	Normal weight pregnant women without gestational diabetes	N/A	Pregnant women who are overweight or have gestational diabetes	N/A	Literature	During pregnancy and birth	Mothers and neonatal care	2011	Payer	No	Modelling	Both overweight mothers (€16,046, %NR) and mothers with gestational diabetes (€13,680, %NR) were associated with higher costs of care.
Xu et al.[13]	2017	Diabetes	China	Pregnant women with gestational diabetes	N/A	Pregnant women without gestational diabetes	N/A	Literature	28 weeks gestation to birth (3 months)	Mothers and neonatal care	2015	Payer	No	Modelling	GDM associated with incremental costs of €1,530 (95%) per case
Meregaglia et al.[14]	2018	Diabetes	Italy	Pregnant women with gestational diabetes	N/A	Pregnant women without gestational diabetes	N/A	Literature	28 weeks gestation to birth (3 months)	Mothers and neonatal care	2014	Payer	No	Modelling	GDM associated with incremental costs of €839 (29%) per case
Mogos et al.[15]	2016	Intimate partner violence	US	Birth-related discharges of women who reported experiencing intimate partner violence	3649	Birth-related discharges of women who did not report experiencing intimate partner violence	32654610	2002-2009	Birth only	Mothers and neonatal care	2006*	Payer	No	Cross-sectional	Intimate partner violence was associated with higher costs of birth-related discharges (€1,410, 33%)
Roberts et al.[16]	2001	Mental Health	Canada	Women who had vaginal births with depression	86	Women who had vaginal births without depression	787	1999	0-4 weeks postpartum	Mothers and children	1999	Payer	No	Cross-sectional	Depression was associated with higher costs (€452, 105%) at 4 weeks postpartum

Author	Year	Morbidity	Setting	Study group	Ns	Comparison group	Nc	Period of data collection	Accrual period for costs	Included costs	Base year for costs	Perspective	Discounting	Study design	Results (absolute cost increase, percentage increase)
Petrou et al.[17]	2002	Mental Health	UK	Primiparous women at high risk of developing postnatal depression who went on to develop it	70	Primiparous women at high risk of developing postnatal depression who did not develop it	136	1997-1999	Birth to 18 weeks postpartum	Mothers and children	2000	Public sector	6%	Cross-sectional	Having postnatal depression was associated with a non-significant increase of €794 (19%) in costs
Dagher et al.[18]	2012	Mental Health	US	Employed mothers with depression	31	Employed mothers without depression	607	2001	From birth to 11 weeks postpartum	Mothers only	2001	Payer	No	Cross-sectional	Depression associated with higher mean expenditure per woman (€576, 186%)
Bauer et al.[19]	2015	Mental Health	UK	Adolescent children (aged 11- 16) of mothers who had perinatal depression	N/A	Adolescent children (aged 11- 16) of mothers who did not have perinatal depression	N/A	Literature	Lifetime of children	Children only	2011	Societal	3.5%	Modelling	Costs associated with exposure to maternal depression were high for the public sector (€4,010, %NR) and for society (€10,838, %NR), which included productivity and HRQoL losses.
Bauer et al.[20]	2016	Mental Health	UK	Mothers with antenatal or postnatal depression or anxiety	N/A	Mothers without antenatal or postnatal depression or anxiety	N/A	Literature	Lifetime of mothers and children	Mothers and children	2013	Societal	3.5%	Modelling	Estimated net present value of per person lifetime costs of depression (€92,642, %NR) and anxiety (€42,586, %NR).
Ammerman et al.[21]	2016	Mental Health	US	Mothers with depression who are at high risk based on social adversity factors (unmarried, Medicaid, low income, etc.)	2298	High-risk mothers without depression	18142	1996-2011	Annual postpartum costs - not pregnancy or birth costs	Mothers only	2011	Societal	No	Cross-sectional	Depression associated with greater probability of incurring expenses (OR 1.51) as well as higher expenditure for those treated, resulting in average incremental cost of €1,564 (55%) per woman
Chojenta et al.[22]	2018	Mental Health	Australia	Babies born to women who had a history of poor mental health (depression/anxiety/other major mental illness)	697	Babies born to women who did not have a history of poor mental health	2987	2002-2011	Pregnancy and 1 year postpartum	Mothers only	2016	Payer	No	Cross-sectional	History of poor mental health is associated with an average increase of €507 (11%) in costs per birth
Moore-Simas et al.[23]	2019	Mental Health	US	Singleton pregnancies in insured women with depression	33,314	Singleton pregnancies in insured women without depression	102,364	2010-2016	24 months postpartum	Children only	2016	Payer	No	Cross sectional	Depression associated with incremental costs of €2,019 (12%) over the first 2 years of the child's life

Author	Year	Morbidity	Setting	Study group	Ns	Comparison group	Nc	Period of data collection	Accrual period for costs	Included costs	Base year for costs	Perspective	Discounting	Study design	Results (absolute cost increase, percentage increase)
Law et al.[24]	2015	Multiple	US	Children of insured mothers without pregnancy-related complications	33725	Children of insured mothers with pregnancy-related complications	103315	2008-2011	First 3 months of life	Children only	2011	Payer	No	Cross-sectional	Higher unadjusted cost of newborn care among mothers with hypertension (€8,174, 112%), obesity (€3,028, 38%), epilepsy (€1,694, 21%) and diabetes (€1,132, 14%)
Law et al.[25]	2015	Multiple	US	Insured mothers without pregnancy-related complications	170941	Insured mothers with pregnancy-related complications	151200	2008-2011	During pregnancy and 3 months postpartum	Mothers only	2011	Payer	No	Cross-sectional	Higher unadjusted cost of maternal care for those with hypertension (€5,382, 40%), obesity (€4,802, 35%), epilepsy (€6,033, 44%) and diabetes (€4,000, 30%)
Hao et al.[26]	2019	Multiple	US	Nulliparous singleton mothers with preeclampsia or hypertension	1,424	Nulliparous singleton mothers without preeclampsia or hypertension	712	2010-2015	20 weeks gestation to 6 weeks postpartum (mothers) or 12 months (infants)	Mothers and children	2015	Payer	No	Cross sectional	Preeclampsia was associated with higher costs of care (€22,360, 217%), as was hypertension (€8,595, 83%)
Piwko et al.[27]	2007	Nausea and vomiting	Canada	Pregnant women with mild nausea or vomiting (NVP)	22	Pregnant women with moderate or severe nausea or vomiting (NVP)	117	2002	During pregnancy (per woman-week)	Mothers only	2005	Societal	No	Cross-sectional	Moderate (€194, 169%) and severe (€454, 395%) NVP was associated with higher costs per women week compared with mild NVP
Piwko et al.[28]	2013	Nausea and vomiting	US	Pregnant women with mild nausea or vomiting (NVP)	N/A	Pregnant women with moderate or severe nausea or vomiting (NVP)	N/A	Literature	During pregnancy (per woman)	Mothers only	2012	Payer (societal costs could not be extracted)	No	Modelling	Higher treatment costs for moderate (€15, 43%) and severe NVP (€191, 568%) compared to mild NVP
Denison et al.[29]	2009	Obesity	UK	Normal weight pregnant women with minor complications	375	Overweight and obese pregnant women with minor complications	276	2007-2008	10-12 weeks gestation to birth	Mothers only	2008	Payer	No	Cross-sectional	Mean additional cost associated with treating minor complications for overweight (€3, 14%) and obese(€49, 215%) mothers
Trasande et al.[30]	2009	Obesity	US	Hospitalisations of pregnant women with a secondary diagnosis of obesity	230689	Hospitalisations of pregnant women without a secondary diagnosis of obesity	1626	1999-2005	Per pregnancy-related episode of care	Mothers only	2005	Payer	No	Cross-sectional	A secondary diagnosis of obesity was associated with higher average costs across all pregnancy-related hospitalisations (€2,643, %NR), and also after adjusting for rate of CS (€1,998, %NR)
Watson et al.[31]	2013	Obesity	Australia	Normal weight singleton mothers (BMI 18.5 - <25)	17175	Overweight (BMI 25 to >30), Obese I (BMI 30 to <35), Obese II (BMI35 to <40), Obese III (BMI ≥40)	19156	2008	During pregnancy to 90 days postpartum	Mothers only	2008	Payer	No	Cross-sectional	Higher mean costs for overweight (€337, 6%), obese I (€609, 11%), obese II (€959, 17%), and obese III (€1,216, 22%) mothers, compared to normal weight mothers

Author	Year	Morbidity	Setting	Study group	Ns	Comparison group	Nc	Period of data collection	Accrual period for costs	Included costs	Base year for costs	Perspective	Discounting	Study design	Results (absolute cost increase, percentage increase)
Denison et al.[32]	2014	Obesity	UK	Normal weight mothers	61232	Mothers who were overweight (BMI2 25-29), obese (BMI 30-39) or severely obese (BMI ≥40)	59441	2003-2010	During pregnancy and birth	Mothers only	2007*	Payer	No	Cross-sectional	Being overweight (€269, 8%), obese (€711, 21%) and severely obese (€1,299, 39%) were associated with higher costs compared to normal weight mothers
Morgan et al.[33]	2014	Obesity	UK	Normal weight mothers (BMI <25)	260	Mothers who were overweight or obese (BMI ≥25)	224	2011-2012	During pregnancy and 2 months postpartum	Mothers only	2012	Payer	No	Cross-sectional	Significantly higher costs associated with obese women (€1,500, 33%), higher mean costs for overweight mothers (€894, 20%) were not statistically significant
Morgan et al.[34]	2015	Obesity	UK	Singleton infants with a normal weight mother	342	Singleton infants with an overweight or obese mother	267	2012-2013	12 months postpartum	Children only	2013	Payer	No	Cross-sectional	Average annual care costs were higher in infants of overweight (€79, 4%) and obese (€1,392, 72%) mothers
Caldas et al.[35]	2015	Obesity	US	Normal weight mothers (BMI <25)	85	Mothers who were class 3 obese (BMI ≥40)	82	2009	During pregnancy and birth	Mothers and children	2009	Payer	No	Cross-sectional	Obesity associated with higher maternity and childcare costs (€10,071, 37%, including hospital and physician costs)
Kuhle et al.[36]	2018	Obesity	Canada	Children of mothers with pre-pregnancy overweight or obesity	10479	Children of mothers without pre-pregnancy obesity	24341	1989-2014	First 18 years of life	Children only	2014	Payer	No	Longitudinal	Having an overweight (€226, %NR) or obese (€1,160, %NR) mother was associated with higher costs of care over the first 18 years of a child’s life
Solmi et al.[37]	2018	Obesity	UK	Normal weight mother with singleton births	4897	Overweight or obese mothers with singleton births	2194	2000-2002	Birth only	Mothers only	2001*	Payer	No	Cross-sectional	Higher unadjusted cost among mothers who were overweight (BMI 25-29: €45, 3%), obese level 1 (BMI 30-34: €165, 11%), and obese level 2/3 (BMI ≥35: €254, 17%)
Fox et al.[38]	2017	Preeclampsia	Ireland	Nulliparous singleton mothers without preeclampsia	166	Nulliparous singleton mothers with preeclampsia	67	2008-2011	15 weeks gestation to 12 months postpartum	Mothers and neonatal care	2016	Payer	No	Cross-sectional	Women with preeclampsia were associated with higher costs of care (€2,860, 114%)

References

1. Chen, Y., et al., *Cost of gestational diabetes mellitus in the United States in 2007.* Popul Health Manag, 2009. **12**(3): p. 165-74.
2. Kolu, P., J. Raitanen, and R. Luoto, *Cost of gestational diabetes-related antenatal visits in health care based on the Finnish Medical Birth Register.* Prim Care Diabetes, 2011. **5**(2): p. 139-41.
3. Cavassini, A.C., et al., *Care cost for pregnant and parturient women with diabetes and mild hyperglycemia.* Rev Saude Publica, 2012. **46**(2): p. 334-43.
4. Kolu, P., et al., *Health care costs associated with gestational diabetes mellitus among high-risk women--results from a randomised trial.* BMC Pregnancy Childbirth, 2012. **12**: p. 71.
5. Anderberg, E., K.S. Carlsson, and K. Berntorp, *Use of healthcare resources after gestational diabetes mellitus: a longitudinal case-control analysis.* Scand J Public Health, 2012. **40**(4): p. 385-90.
6. Gillespie, P., et al., *Modeling the independent effects of gestational diabetes mellitus on maternity care and costs.* 2013. **36**(5): p. 1111-1116.
7. Son, K.H., et al., *Comparison of maternal morbidity and medical costs during pregnancy and delivery between patients with gestational diabetes and patients with pre-existing diabetes.* Diabet Med, 2015. **32**(4): p. 477-86.
8. Dall, T.M., et al., *The economic burden of elevated blood glucose levels in 2012: diagnosed and undiagnosed diabetes, gestational diabetes mellitus, and prediabetes.* Diabetes Care, 2014. **37**(12): p. 3172-9.
9. Danyliv, A., et al., *Short- and long-term effects of gestational diabetes mellitus on healthcare cost: a cross-sectional comparative study in the ATLANTIC DIP cohort.* Diabet Med, 2015. **32**(4): p. 467-76.
10. Jovanovic, L., et al., *Trends in the incidence of diabetes, its clinical sequelae, and associated costs in pregnancy.* Diabetes Metab Res Rev, 2015. **31**(7): p. 707-16.
11. Whiteman, V.E., et al., *Additive effects of Pre-pregnancy body mass index and gestational diabetes on health outcomes and costs.* Obesity (Silver Spring), 2015. **23**(11): p. 2299-308.
12. Lenoir-Wijnkoop, I., et al., *Health economic modeling to assess short-term costs of maternal overweight, gestational diabetes, and related macrosomia - a pilot evaluation.* Frontiers in Pharmacology, 2015. **6**(MAY).
13. Xu, T., et al., *The short-term health and economic burden of gestational diabetes mellitus in China: a modelling study.* BMJ Open, 2017. **7**(12): p. e018893.
14. Meregaglia, M., et al., *The short-term economic burden of gestational diabetes mellitus in Italy.* BMC Pregnancy Childbirth, 2018. **18**(1): p. 58.
15. Mogos, M.F., et al., *The Feto-Maternal Health Cost of Intimate Partner Violence Among Delivery-Related Discharges in the United States, 2002-2009.* J Interpers Violence, 2016. **31**(3): p. 444-64.
16. Roberts, J., et al., *Costs of postpartum care: examining associations from the Ontario mother and infant survey.* Can J Nurs Res, 2001. **33**(1): p. 19-34.
17. Petrou, S., et al., *Economic costs of post-natal depression in a high-risk British cohort.* Br J Psychiatry, 2002. **181**: p. 505-12.
18. Dagher, R.K., et al., *Postpartum depression and health services expenditures among employed women.* J Occup Environ Med, 2012. **54**(2): p. 210-5.
19. Bauer, A., et al., *Perinatal depression and child development: exploring the economic consequences from a South London cohort.* Psychol Med, 2015. **45**(1): p. 51-61.
20. Bauer, A., M. Knapp, and M. Parsonage, *Lifetime costs of perinatal anxiety and depression.* J Affect Disord, 2016. **192**: p. 83-90.
21. Ammerman, R.T., et al., *Annual direct health care expenditures and employee absenteeism costs in high-risk, low-income mothers with major depression.* Journal of Affective Disorders, 2016. **190**: p. 386-394.
22. Chojenta, C., et al., *The impact of a history of poor mental health on health care costs in the perinatal period.* Archives of Women's Mental Health, 2018.
23. Moore Simas, T.A., et al., *Matched cohort study of healthcare resource utilization and costs in young children of mothers with postpartum depression in the United States.* Journal of Medical Economics, 2019.
24. Law, A., et al., *Costs of Newborn Care Following Complications During Pregnancy and Delivery.* Matern Child Health J, 2015. **19**(9): p. 2081-8.
25. Law, A., et al., *The prevalence of complications and healthcare costs during pregnancy.* J Med Econ, 2015. **18**(7): p. 533-41.
26. Hao, J., et al., *Maternal and Infant Health Care Costs Related to Preeclampsia.* Obstetrics and gynecology, 2019.
27. Piwko, C., et al., *The weekly cost of nausea and vomiting of pregnancy for women calling the Toronto Motherisk Program.* Curr Med Res Opin, 2007. **23**(4): p. 833-40.
28. Piwko, C., et al., *Economic burden of nausea and vomiting of pregnancy in the USA.* J Popul Ther Clin Pharmacol, 2013. **20**(2): p. e149-60.
29. Denison, F.C., et al., *Increased maternal BMI is associated with an increased risk of minor complications during pregnancy with consequent cost implications.* Bjog, 2009. **116**(11): p. 1467-72.
30. Trasande, L., et al., *Incremental charges, costs, and length of stay associated with obesity as a secondary diagnosis among pregnant women.* Med Care, 2009. **47**(10): p. 1046-52.
31. Watson, M., et al., *Pre-pregnancy BMI: costs associated with maternal underweight and obesity in Queensland.* Aust N Z J Obstet Gynaecol, 2013. **53**(3): p. 243-9.
32. Denison, F.C., et al., *Association between maternal body mass index during pregnancy, short-term morbidity, and increased health service costs: a population-based study.* Bjog, 2014. **121**(1): p. 72-81; discussion 82.
33. Morgan, K.L., et al., *Obesity in pregnancy: a retrospective prevalence-based study on health service utilisation and costs on the NHS.* BMJ Open, 2014. **4**(2): p. e003983.
34. Morgan, K.L., et al., *Obesity in pregnancy: infant health service utilisation and costs on the NHS.* BMJ Open, 2015. **5**(11): p. e008357.
35. Caldas, M.C., et al., *Maternal morbid obesity: financial implications of weight management.* Clin Obes, 2015. **5**(6): p. 333-41.
36. Kuhle, S., et al., *Maternal pre-pregnancy obesity and health care utilization and costs in the offspring.* International Journal of Obesity, 2018: p. 1-9.
37. Solmi, F. and S. Morris, *Overweight and obese pre-pregnancy BMI is associated with higher hospital costs of childbirth in England.* BMC Pregnancy Childbirth, 2018. **18**(1): p. 253.
38. Fox, A., et al., *Estimating the Cost of Preeclampsia in the Healthcare System: Cross-Sectional Study Using Data From SCOPE Study (Screening for Pregnancy End Points).* Hypertension, 2017. **70**(6): p. 1243-1249.
