# Supplementary material for: Economic burden of maternal morbidity – A systematic review of cost-of-illness studies
Source: PLoS One. 2020 Jan 16;15(1):e0227377. doi: 10.1371/journal.pone.0227377 (PMC6964978; doi:10.1371/journal.pone.0227377)
Supplement: S3 Table — (PDF) [file pone.0227377.s004.pdf]

S3 Table – Results of quality appraisal

Author	Was the study question well specified?	Were quantification methods appropriate and well-executed?	Were healthcare resources valued appropriately?	Was the inclusion of intangible costs appropriate?	Was a range of estimates presented?	Was a sensitivity analysis performed?	Was uncertainty around the estimates and its implications adequately discussed?	Were important limitations discussed regarding the cost components, data, assumptions and methods?	Will the results help to understand costs of maternal morbidity?
Chen et al.[1]	Yes	Yes	Yes	Not applicable	No	No	No	Yes	Yes
Kolu et al.[2]	Yes	Yes	Yes	Not applicable	No	No	No	No	Yes
Cavassini et al.[3]	Yes	Yes	Yes	Not applicable	No	No	No	No	Yes
Kolu et al.[4]	Yes	Yes	Yes	No	No	No	No	Yes	Yes
Anderberg et al.[5]	Yes	Yes	Yes	Not applicable	No	No	No	Yes	Yes
Gillespie et al.[6]	Yes	Yes	Yes	Not applicable	Yes	Yes	Yes	Yes	Yes
Son et al.[7]	Yes	Yes	Yes	Not applicable	No	No	Yes	Yes	Yes
Dall et al.[8]	Yes	Yes	Yes	Yes	No	No	No	Yes	Yes
Danyliv et al.[9]	Yes	Yes	Yes	Not applicable	Yes	No	No	Yes	Yes
Jovanovic et al.[10]	Yes	Yes	Yes	Not applicable	No	No	No	Yes	Yes
Whiteman et al.[11]	Yes	Yes	Yes	Not applicable	No	No	No	Yes	Yes
Lenoir-Wijnkoop et al.[12]	Yes	Yes	Can't say	Not applicable	No	Yes	No	Yes	Yes
Xu et al.[13]	Yes	Yes	Yes	Not applicable	No	Yes	Yes	Yes	Yes
Meregaglia et al.[14]	Yes	Yes	Yes	Not applicable	No	Yes	Yes	Yes	Yes
Mogos et al.[15]	Yes	Yes	Yes	Not applicable	No	Yes	Yes	Yes	Yes
Roberts et al.[16]	Yes	Yes	Yes	No	No	No	No	Yes	Yes
Petrou et al.[17]	Yes	Yes	Yes	Not applicable	Yes	Yes	Yes	Yes	Yes
Dagher et al.[18]	Yes	Yes	Yes	Not applicable	Yes	No	No	Yes	Yes
Bauer et al.[19]	Yes	Yes	Yes	Yes	No	No	No	Yes	Yes
Bauer et al.[20]	Yes	Yes	Yes	Yes	No	No	No	Yes	Yes
Ammerman et al.[21]	Yes	Yes	Yes	Yes	No	Yes	Yes	Yes	Yes
Chojenta et al.[22]	Yes	Yes	Yes	Not applicable	Yes	No	No	Yes	Yes
Law et al.[23]	Yes	Yes	Yes	Not applicable	Yes	No	No	Yes	Yes
Law et al.[24]	Yes	Yes	Yes	Not applicable	Yes	Yes	Yes	Yes	Yes
Piwko et al.[25]	Yes	Yes	Yes	Yes	No	No	No	No	Yes
Piwko et al.[26]	Yes	Yes	Can't say	Not applicable *	No	No	Yes	No	Yes
Denison et al.[27]	Yes	Yes	Yes	Not applicable	No	No	No	Yes	Yes
Trasande et al.[28]	Yes	Yes	Yes	Not applicable	Yes	No	Yes	No	Yes
Watson et al.[29]	Yes	Yes	Yes	Not applicable	No	No	No	Yes	Yes
Denison et al.[30]	Yes	Yes	Yes	Not applicable	Yes	Yes	Yes	Yes	Yes
Morgan et al.[31]	Yes	Yes	Yes	Not applicable	Yes	No	Yes	Yes	Yes
Morgan et al.[32]	Yes	Yes	Yes	Not applicable	Yes	No	No	Yes	Yes
Caldas et al.[33]	Yes	Yes	Yes	Not applicable	No	No	No	Yes	Yes
Kuhle et al.[34]	Yes	Yes	Can't say	Not applicable	No	No	No	Yes	Yes
Solmi et al.[35]	Yes	Yes	Yes	Not applicable	Yes	Yes	No	Yes	Yes
Fox et al.[36]	Yes	Yes	Yes	Not applicable	No	Yes	No	Yes	Yes
Hao et al.[37]	Yes	Yes	Yes	Not applicable	No	No	No	Yes	Yes
Moore-Simas et al.[38]	Yes	Yes	Yes	Not applicable	Yes	Yes	No	Yes	Yes

Key: Yes (green) No (red) Can't say (yellow) Not applicable (grey) * This study adopted a societal perspective but only costs from the payer perspective could be extracted

References

1. Chen, Y., et al., *Cost of gestational diabetes mellitus in the United States in 2007.* Popul Health Manag, 2009. **12**(3): p. 165-74.
2. Kolu, P., J. Raitanen, and R. Luoto, *Cost of gestational diabetes-related antenatal visits in health care based on the Finnish Medical Birth Register.* Prim Care Diabetes, 2011. **5**(2): p. 139-41.
3. Cavassini, A.C., et al., *Care cost for pregnant and parturient women with diabetes and mild hyperglycemia.* Rev Saude Publica, 2012. **46**(2): p. 334-43.
4. Kolu, P., et al., *Health care costs associated with gestational diabetes mellitus among high-risk women--results from a randomised trial.* BMC Pregnancy Childbirth, 2012. **12**: p. 71.
5. Anderberg, E., K.S. Carlsson, and K. Berntorp, *Use of healthcare resources after gestational diabetes mellitus: a longitudinal case-control analysis.* Scand J Public Health, 2012. **40**(4): p. 385-90.
6. Gillespie, P., et al., *Modeling the independent effects of gestational diabetes mellitus on maternity care and costs.* 2013. **36**(5): p. 1111-1116.
7. Son, K.H., et al., *Comparison of maternal morbidity and medical costs during pregnancy and delivery between patients with gestational diabetes and patients with pre-existing diabetes.* Diabet Med, 2015. **32**(4): p. 477-86.
8. Dall, T.M., et al., *The economic burden of elevated blood glucose levels in 2012: diagnosed and undiagnosed diabetes, gestational diabetes mellitus, and prediabetes.* Diabetes Care, 2014. **37**(12): p. 3172-9.
9. Danyliv, A., et al., *Short- and long-term effects of gestational diabetes mellitus on healthcare cost: a cross-sectional comparative study in the ATLANTIC DIP cohort.* Diabet Med, 2015. **32**(4): p. 467-76.
10. Jovanovic, L., et al., *Trends in the incidence of diabetes, its clinical sequelae, and associated costs in pregnancy.* Diabetes Metab Res Rev, 2015. **31**(7): p. 707-16.
11. Whiteman, V.E., et al., *Additive effects of Pre-pregnancy body mass index and gestational diabetes on health outcomes and costs.* Obesity (Silver Spring), 2015. **23**(11): p. 2299-308.
12. Lenoir-Wijnkoop, I., et al., *Health economic modeling to assess short-term costs of maternal overweight, gestational diabetes, and related macrosomia - a pilot evaluation.* Frontiers in Pharmacology, 2015. **6**(MAY).
13. Xu, T., et al., *The short-term health and economic burden of gestational diabetes mellitus in China: a modelling study.* BMJ Open, 2017. **7**(12): p. e018893.
14. Meregaglia, M., et al., *The short-term economic burden of gestational diabetes mellitus in Italy.* BMC Pregnancy Childbirth, 2018. **18**(1): p. 58.
15. Mogos, M.F., et al., *The Feto-Maternal Health Cost of Intimate Partner Violence Among Delivery-Related Discharges in the United States, 2002-2009.* J Interpers Violence, 2016. **31**(3): p. 444-64.
16. Roberts, J., et al., *Costs of postpartum care: examining associations from the Ontario mother and infant survey.* Can J Nurs Res, 2001. **33**(1): p. 19-34.
17. Petrou, S., et al., *Economic costs of post-natal depression in a high-risk British cohort.* Br J Psychiatry, 2002. **181**: p. 505-12.
18. Dagher, R.K., et al., *Postpartum depression and health services expenditures among employed women.* J Occup Environ Med, 2012. **54**(2): p. 210-5.
19. Bauer, A., et al., *Perinatal depression and child development: exploring the economic consequences from a South London cohort.* Psychol Med, 2015. **45**(1): p. 51-61.
20. Bauer, A., M. Knapp, and M. Parsonage, *Lifetime costs of perinatal anxiety and depression.* J Affect Disord, 2016. **192**: p. 83-90.
21. Ammerman, R.T., et al., *Annual direct health care expenditures and employee absenteeism costs in high-risk, low-income mothers with major depression.* Journal of Affective Disorders, 2016. **190**: p. 386-394.
22. Chojenta, C., et al., *The impact of a history of poor mental health on health care costs in the perinatal period.* Archives of Women's Mental Health, 2018.
23. Law, A., et al., *Costs of Newborn Care Following Complications During Pregnancy and Delivery.* Matern Child Health J, 2015. **19**(9): p. 2081-8.
24. Law, A., et al., *The prevalence of complications and healthcare costs during pregnancy.* J Med Econ, 2015. **18**(7): p. 533-41.
25. Piwko, C., et al., *The weekly cost of nausea and vomiting of pregnancy for women calling the Toronto Motherisk Program.* Curr Med Res Opin, 2007. **23**(4): p. 833-40.
26. Piwko, C., et al., *Economic burden of nausea and vomiting of pregnancy in the USA.* J Popul Ther Clin Pharmacol, 2013. **20**(2): p. e149-60.
27. Denison, F.C., et al., *Increased maternal BMI is associated with an increased risk of minor complications during pregnancy with consequent cost implications.* Bjog, 2009. **116**(11): p. 1467-72.
28. Trasande, L., et al., *Incremental charges, costs, and length of stay associated with obesity as a secondary diagnosis among pregnant women.* Med Care, 2009. **47**(10): p. 1046-52.
29. Watson, M., et al., *Pre-pregnancy BMI: costs associated with maternal underweight and obesity in Queensland.* Aust N Z J Obstet Gynaecol, 2013. **53**(3): p. 243-9.
30. Denison, F.C., et al., *Association between maternal body mass index during pregnancy, short-term morbidity, and increased health service costs: a population-based study.* Bjog, 2014. **121**(1): p. 72-81; discussion 82.
31. Morgan, K.L., et al., *Obesity in pregnancy: a retrospective prevalence-based study on health service utilisation and costs on the NHS.* BMJ Open, 2014. **4**(2): p. e003983.
32. Morgan, K.L., et al., *Obesity in pregnancy: infant health service utilisation and costs on the NHS.* BMJ Open, 2015. **5**(11): p. e008357.
33. Caldas, M.C., et al., *Maternal morbid obesity: financial implications of weight management.* Clin Obes, 2015. **5**(6): p. 333-41.
34. Kuhle, S., et al., *Maternal pre-pregnancy obesity and health care utilization and costs in the offspring.* International Journal of Obesity, 2018: p. 1-9.
35. Solmi, F. and S. Morris, *Overweight and obese pre-pregnancy BMI is associated with higher hospital costs of childbirth in England.* BMC Pregnancy Childbirth, 2018. **18**(1): p. 253.
36. Fox, A., et al., *Estimating the Cost of Preeclampsia in the Healthcare System: Cross-Sectional Study Using Data From SCOPE Study (Screening for Pregnancy End Points).* Hypertension, 2017. **70**(6): p. 1243-1249.
37. Hao, J., et al., *Maternal and Infant Health Care Costs Related to Preeclampsia.* Obstetrics and gynecology, 2019.
38. Moore Simas, T.A., et al., *Matched cohort study of healthcare resource utilization and costs in young children of mothers with postpartum depression in the United States.* Journal of Medical Economics, 2019.
